# Concomitant Administration of Rosuvastatin and Lefleunamide in Low doses Synergize Against Complete Freunds Adjuvant (CFA)-Induced Rheumatoid Arthritis in Experimental Model

**DOI:** 10.9734/jpri/2021/v33i42a32415

**Published:** 2021-08-30

**Authors:** Abdel-Aziz Saeed, Mohamed El-Shafey, Gouda K. Helal, El-Sayed Akool

**Affiliations:** 1 Pharmacology and Toxicology Department, Faculty of Pharmacy, Al-Azhar University, Cairo, Egypt; 2 Pharmacology and Toxicology Department, Faculty of Pharmacy, Heliopolis University, Cairo, Egypt

**Keywords:** Rosuvastatin, lefleunamide, rheumatoid arthritis, complete freunds adjuvant

## Abstract

**Aim:**

The present work was designed to examine of the potential anti-inflammatory effect of rosuvastatin (ROSV) and/or Lefleunamide (LFLU) against Complete Freunds Adjuvant (CFA)-induced arthritis in rats.

**Methods:**

The mRNA level of perxisome proliferator-activated receptor-alpha (PPAR-α) was determined using Real-time PCR. The levels of NF-κB, iNOS, IL-6, TNF-α and SOD activity were measured using ELISA. The swollen paws were measured using caliper. The GSH level was measured using colorimetric assay. The level of malondialdehyde (MDA) was determined using thiobarbituric acid reactive substances assay kit.

**Results:**

ROSV induced the expression of PPAR-α that suppresses NF-κB as demonstrated by a strong reduction in NF-κB level in animals treated with ROSV. Also, ROSV administration reduced the levels of the inflammatory mediators IL-6 and TNF-α. In addition, iNOS and MDA content as well as expression of MMP-9 and MMP-2 induced by CFA is abrogated in animals treated with ROSV. Also GSH content and SOD activity were highly increased in ROSV-treated animals. Furthermore, the size of right paw induced by CFA was reduced in ROSV-treated rats. Moreover, the histopathological alterations induced by CFA were highly improved in animals treated with ROSV. Similar results were also found in animals treated with LFLU. Importantly, similar effects were obtained in rats treated with both ROSV and LFLU in half doses.

**Conclusion:**

This study demonstrates that ROSV as well as LFLU has the ability to inhibit rheumatoid arthritis in experimental model induced by CFA. Importantly, concomitant administration of ROSV and LFLU in half doses synergize against rheumatoid arthritis.

## 1. INTRODUCTION

Rheumatoid arthritis (RA) is a chronic disease manifested by joint pain, tenderness and swelling [1]. Many studies reported the role of autoimmune phenomenon in the development of RA [2,3]. Activation of B-cells has been shown to release IgM antibody against IgG; this molecule is called rheumatoid factor (RF). The immune complexes (IgG and IgM) trigger inflammatory destruction to the synovium and collagen [2,3]. It has been shown that macrophages activation releases several cytokines which play an important role in joint tissues damage [2,3]. The destruction of the cartilage has been shown to be due to matrix metalloproteinases (MMPs) activity, produced by activated macrophages and fibroblasts in response to inflammatory cytokines such as interleukin-1 (IL-1) and tumor necrosis factor-α (TNF-α) [4]. Nuclear Factor Kappa B (NF-κB) is highly activated at sites of inflammation in variety of diseases and can control the production of proinflammatory cytokines, MMPs, adhesion molecules, inducible nitric oxide synthase (iNOS) and cyclooxygenase-2 (Cox-2) [5]. The peroxisome proliferator-activated receptors (PPARs) form a subfamily of the nuclear receptor superfamily. There are three isoforms: PPAR γ, PPAR α, and PPAR δ. The PPARs are ligand dependent transcription factors that control the transcription of different target genes through the binding to specific peroxisome proliferator response elements (PPREs) in enhancer sites of regulated genes. PPARs are expressed in immunological cell types such as monocyte/macrophages, lymphocyte, and dendritic cells. The three isoforms have been shown to inhibit the production of many inflammatory mediators and cytokines [6]. PPAR-α agonists have been shown to modulate inflammation by inhibiting cytokine (TNF-α, interleukins) production in a PPAR-α dependent manner [7–10]. Statins have been shown to induce PPAR-α expression in stimulated endothelial cells, macrophages, and hepatocytes [11]. Several drugs are usually used to manage RA like analgesics, disease-modifying anti-rheumatic drugs (DMARDs), non-steroidal anti-inflammatory drugs (NSAIDs), corticosteroids and immunosuppressive agents. Also, TNF-α and IL-1β antagonists have been used in management of RA patients [12,13]. However, the uses of these agents are usually associated with several adverse effects. Recently, researchers are directed towards the discovery of safe and effective drugs for the long-term use. Kleemann and his colleagues [14] reported that, the anti-inflammatory effect of rosuvastatin (ROSV) is mediated via peroxisome proliferator-activated receptors (PPARs) signaling-pathway through suppression of NF-κB mediated-target gene activation especially TNF-α, IL-6, adhesion molecules and iNOS. Furthermore, it has been reported that combination of atrovastatin with prednisolone produced a better results than in either remedy alone against Freunds adjuvant induced-arthritis in rats [15]. Interestingly, it has been demonstrated that inhibitors of HMG-CoA reductase may protect joints and peri-articular bones of experimental animals against experimental arthritis progression [16]. Leflunomide (LFLU) which belongs to DMARD was licensed for use in rheumatoid arthritis in 1998. It interferes with the production of inflammatory cytokines by T-cell via inhibition of NF-κB activity required for inflammatory cytokines expression [17]. It inhibits also the production of proinflammatory TNF-α and interleukin 1β [18]. In addition, LFLU has the ability to inhibit COX-2 enzyme at the site of inflammation [19]. Therefore, the present work was designed to examine first, the potential antiinflammatory effect of either ROSV or LFLU [standard DMARD] against Complete Freunds Adjuvant (CFA)-induced arthritis in rats. Second the potential anti-inflammatory effect of both ROSV and LFLU when given together in half doses against CFA-induced arthritis in rats.

## 2. MATERIALS AND METHODS

### 2.1 Animals

Female Wistar albino rats weighing 150-200 g were provided from Nile Co., Cairo, Egypt. The rats were housed in a 12h dark/light cycle animal facility with controlled humidity and constant temperature. A standard diet and water were supplied ad libitum. For adaptation, the rats were kept for one week under observation before the experimental study.

### 2.2 Materials

Complete Freunds Adjuvant (CFA) was purchased from Sigma-Aldrich Co. (St. Louis, MO, USA). Rosuvastatin was obtained from AstraZeneca Pharmaceuticals, Egypt. Lefleunamide was obtained from Multi Apex Pharmaceuticals, Egypt. Reduced glutathione (GSH), superoxide dismutase (SOD), and thiobarbituric acid reactive substances (TBARS) assay kits were purchased from Bio-diagnostic Co. (Giza, Egypt). Tumor necrosis factor-α (TNF-α) and interleukin-6 (IL-6) ELISA kits were purchased from Abcam Inc., (Cambridge, MA, USA). Inducible NO-synthase (iNOS) was purchased from EIAab Science Co., Ltd., China. Nuclear factor kappa-B (NF-κB), matrix-metalloproteinase-2 (MMP-2) and matrix-metalloproteinase-9 (MMP-9) were purchased from Cloud-Clone Corp., Houston, TX, USA.

### 2.3 Experimental Design

The rats were randomly divided into five groups, 8 rats in each. The control group (first group) was given the vehicle (0.5% Sodium carboxymethyl cellulose). The second group received 0.4ml CFA (SC) in right hind paw for 12 days divided in three doses to induce rheumatoid arthritis [20]. After induction of rheumatoid arthritis for 12 days, the third group were administered ROSV on day 13 in a dose of 10 mg/kg/day [21] for 28 days after the last injection of CFA. Also, after induction of rheumatoid arthritis for 12 days, the fourth group received LFLU on day 13 in a dose of 10 mg/kg/day [22] for 28 days after the last injection of CFA. On day 13, after induction of rheumatoid arthritis for 12 days, the fifth group was treated with ROSV in a dose of 5 mg/kg/day [23] plus LFLU in a dose of 5 mg/kg/day [24,25] for 28 days after the last injection of CFA. At the end, blood was collected for measurement of IL-6 and TNF-α levels. Afterwards, animals were sacrificed by cervical dislocation. Ankle, paw and knee joints were dissected immediately after death, washed with ice-cold phosphate buffered saline (PBS), and kept at −20°C for biochemical analysis. Paws specimens were kept in 10% neutral-buffered formal saline for histopathological analysis.

### 2.4 Assessment of NF-κB Level

The NF-κB level in joint tissue was assessed by enzyme-linked immunosorbent assay (ELISA) as previously described [26].

### 2.5 Assessment of iNOS Level

The iNOS level in joint tissue was detected by enzyme-linked immunosorbent assay (ELISA) according to the manufacturers instructions (EIAab Science Co.,Ltd., China).

### 2.6 Determination of Serum IL-6 Level

The serum level of IL-6 in was measured by enzyme-linked immunosorbent assay (ELISA) according to the manufacturers instructions (Abcam Inc., Cambridge, MA, USA).

### 2.7 Determination of Serum TNF-α Level

The serum level of TNF-α was detected by enzyme-linked immunosorbent assay (ELISA) according to the manufacturers instructions (Abcam Inc., Cambridge, MA, USA).

### 2.9 Measurement of Lipid Peroxides

The level of malondialdehyde (MDA) content in joint tissue was assessed according to the manufacturers instructions (Bio-diagnostic Co., Giza, Egypt) as previously described [26]. Briefly, the determination of TBARS calculated as MDA is based on the reaction of MDA with TBARS. The absorbance of the resultant pink color was determined at 534 nm spectrophotometrically. The MDA content in joint tissue samples was determined by comparison with the predetermined MDA standard curve.

### 2.10 Measurement of SOD Activity

The SOD activity in joint tissue was determined as previously described [27].

### 2.11 Assessment of GSH Content

The GSH content in joint tissues was assessed as previously described [26]. Briefly, GSH determination depends on the reduction of Ellmans reagent by the SH group in GSH to produce yellow product which can be measured spectrophotometrically at 405 nm.

### 2.12 Real-time PCR

The level of PPAR-α mRNA in joint tissue was detected using Real-time PCR system as previously described [28]. Briefly, total RNA was extracted from joint tissue according to the manufacturers instructions of the total RNA extraction kit and the RNA was subjected to reverse transcription. The following primers were used:

PPAR-α: 5′-ACGATGCTGTCCTCCTTGATG-3′ (forward),

5′- GCGTCTGACTCGGTCTTCTTG-3′ (reverse)

GAPDH: 5′-CCATTCTTCCACCTTTGATGCT-3′ (forward),

5′-TGTTGCTGTAGCCATATTCATTGT-3′ (reverse)

Real time PCR was done as follows: one initial step at 95°C for 10 min followed by 45 cycles at 95°C for 10 seconds, 55°C for 1 min and 72°C extension step for 5 seconds. The mRNA level was determined as fold change from the GAPDH level.

### 2.13 Measurement of Paw Size

The swollen paws were periodically examined (up to 28 days) in each paw from the ankle using caliper as previously described [29].

### 2.14 Histopathological Investigation

Histopathological examination was done as previously described [27].

### 2.15 Statistical Analysis

Results are expressed as means ± SD. One way ANOVA followed by Tukey-Kramer as a post-hoc test was used to analyze statistical significance among groups. P-values below 0.05 were considered as indication for statistically significant differences between groups compared.

## 3. RESULTS

### 3.1 ROSV and/or LFLU Upregulate PPAR-α mRNA Transcription in Joint Tissue of Arthritic Animals

**Fig. 1.**
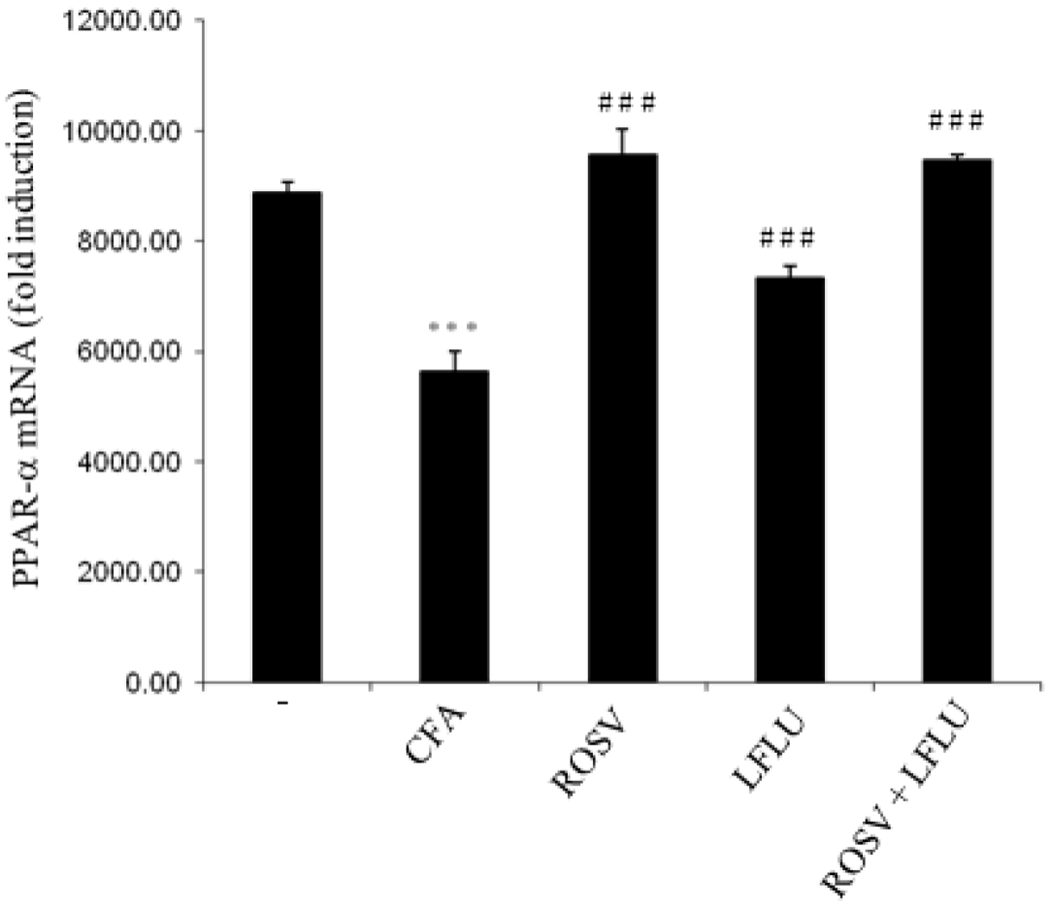
Effect of ROSV and/or LFLU on PPAR-α expression in experimental model of RA induced by CFA Total RNA was isolated from joint tissues of animals treated with either vehicle (−) or CFA (0.4ml Complete Fruends Adjuvant S.C in right hind paw) or CFA+ROSV (10 mg/kg/day) or CFA+LFLU (10 mg/kg/day) or CFA+(ROSVand LFLU in half doses) and mRNA expression of PPAR-α was assessed by Real-time PCR analysis. PPAR-α mRNA was normalized to that of GAPDH and is shown as mean fold-induction. Data represent means ± S.D. (n=8), *** p < 0.001 versus control, ### p < 0.001, versus CFA alone-treated animals

As demonstrated in Fig. 1, induction of rheumatoid arthritis with CFA significantly reduced PPAR-α expression on mRNA level as compared to control. However, treatment of rats with either ROSV or LFLU (standard DMARD) significantly induced PPAR-α expression in joint tissue of arthritic animals. Concomitant administration of ROSV and LFLU in half doses significantly induced PPAR-α expression.

### 3.2 ROSV and/or LFLU Attenuate NF-κB and iNOS Expression in Experimental Model of RA Induced by CFA

**Fig. 2.**
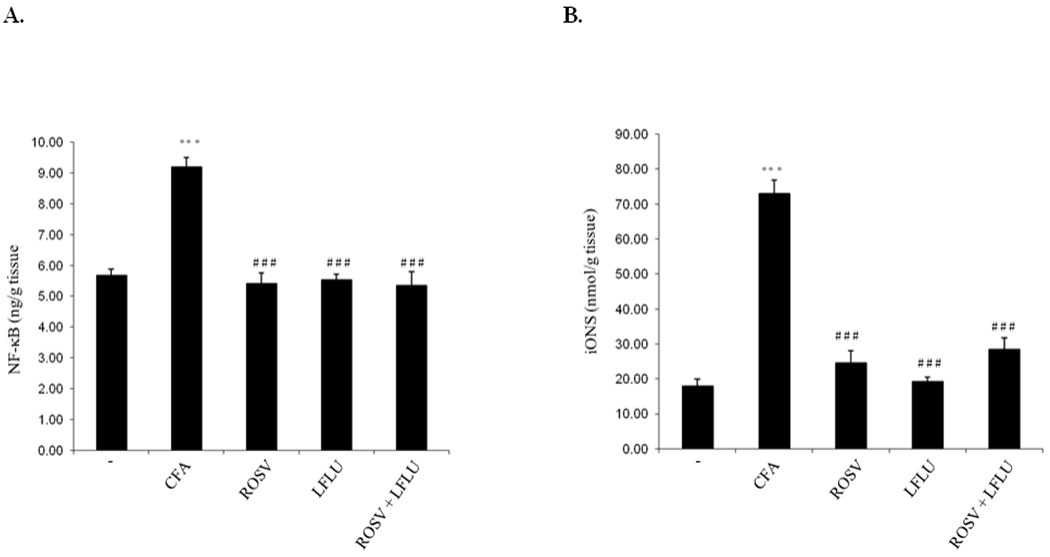
Effect of ROSV and/or LFLU on NF-κB and iNOS expression in experimental model of RA induced by CFA NF-κB (A) and iNOS (B) expression in joint tissue from rats treated with with either vehicle (−) or CFA (0.4ml Complete Fruends Adjuvant S.C in right hind paw) or CFA+ROSV (10 mg/kg/day) or CFA+LFLU (10 mg/kg/day) or CFA+(ROSVand LFLU in half doses) were measured by ELISA. Data represent means ± S.D. (n=8), *** p < 0.001 versus control, ### p < 0.001, versus CFA alone-treated animals

Treatment of rats with either ROSV or LFLU significantly attenuated NF-κB expression induced by CFA in arthritic group (Fig. 2A). In addition, treatment of animals with ROSV in combination with LFLU in half doses significantly reduced NF-κB expression in arthritic animals (Fig. 2A). In addition, iNOS expression was highly reduced in arthritic animals treated with either ROSV or LFLU (Fig. 2B). Also, concomitant use of ROSV and LFLU in half doses significantly inhibited iNOS expression in joint tissue of arthritic rats (Fig. 2B).

### 3.3 ROSV and/or LFLU Downregulate the Expression of MMP-9 and MMP-2 in Joint Tissue of Arthritic Animals

**Fig. 3.**
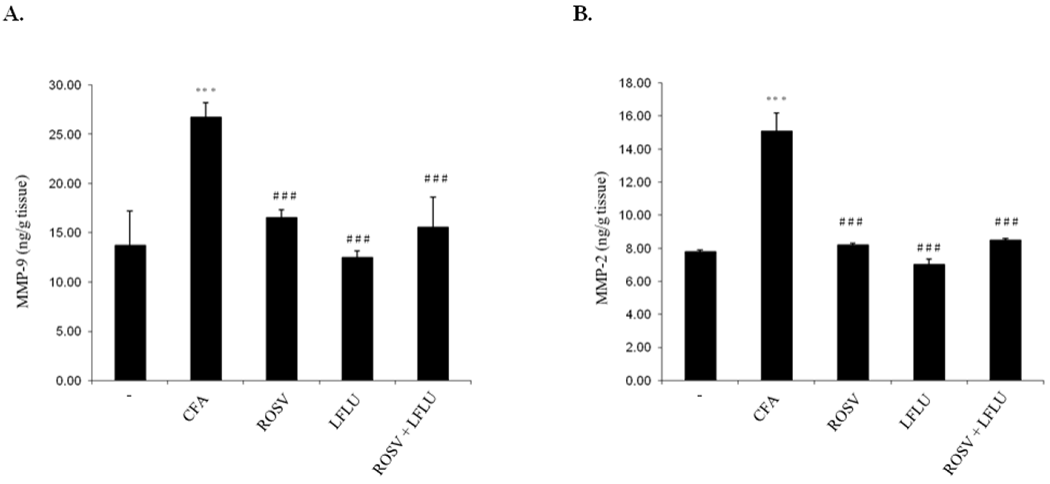
Effect of ROSV and/or LFLU on MMP-9 and MMP-2 expression in experimental model of RA induced by CFA MMP-9 (A) and MMP-2 (B) expression in joint tissue from rats treated with with either vehicle (−) or CFA (0.4ml Complete Fruends Adjuvant S.C in right hind paw) or CFA+ROSV (10 mg/kg/day) or CFA+LFLU (10 mg/kg/day) or CFA+(ROSVand LFLU in half doses) were measured by ELISA. Data represent means ± S.D. (n=8), *** p < 0.001 versus control, ### p < 0.001 versus CFA alone-treated animals

As demonstrated in Fig. 3, the expression of MMP-9 and MMP-2 were highly induced in joint tissue of arthritic animals (Fig. 3A, 3B). On the other hand, treatment of arthritic rats with either ROSV or LFLU significantly inhibited the expression of MMP-9 and MMP-2 in joint tissue of arthritic animals (Fig. 3A, 3B). Concomitant use of ROSV and LFLU in half doses significantly reduced the expression of MMP-9 and MMP-2 in arthritic animals (Fig. 3A, 3B).

### 3.4 ROSV and/or LFLU Attenuate TNF-α and IL-6 Expression in Joint Tissue of Arthritic Animals

**Fig. 4.**
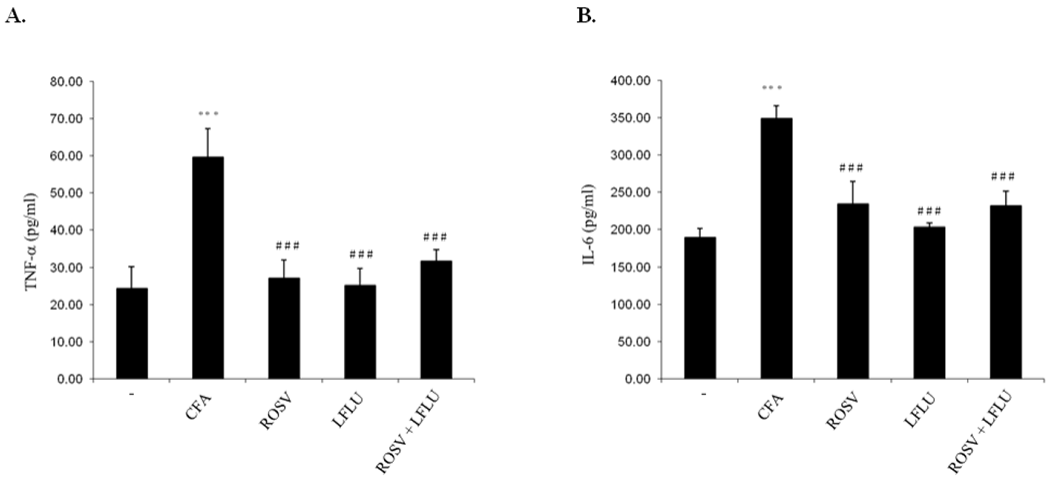
Effect of ROSV and/or LFLU on TNF-α and IL-6 expression in experimental model of RA induced by CFA Serum levels of TNF-α (A) and IL-6 (B) from rats treated with with either vehicle (−) or CFA (0.4ml Complete Fruends Adjuvant S.C in right hind paw) or CFA+ROSV (10 mg/kg/day) or CFA+LFLU (10 mg/kg/day) or CFA+(ROSVand LFLU in half doses) were measured by ELISA. Data represent means ± S.D. (n=8), *** p < 0.001 versus control, ### p < 0.001 versus CFA alone-treated animals

The level of TNF–α and IL-6 were highly increased in serum of arthritic animals (Fig. 4A, 4B). On the other hand, treatment of arthritic rats with either ROSV or LFLU significantly reduced the level of TNF-α and IL-6 (Fig. 4A, 4B). Concomitant use of ROSV and LFLU in half doses significantly reduced the level of TNF-α and IL-6 (Fig 4A, 4B).

### 3.5 ROSV and/or LFLU Inhibit Lipid Peroxidation in Joint Tissue of Arthritic Animals

**Fig. 5.**
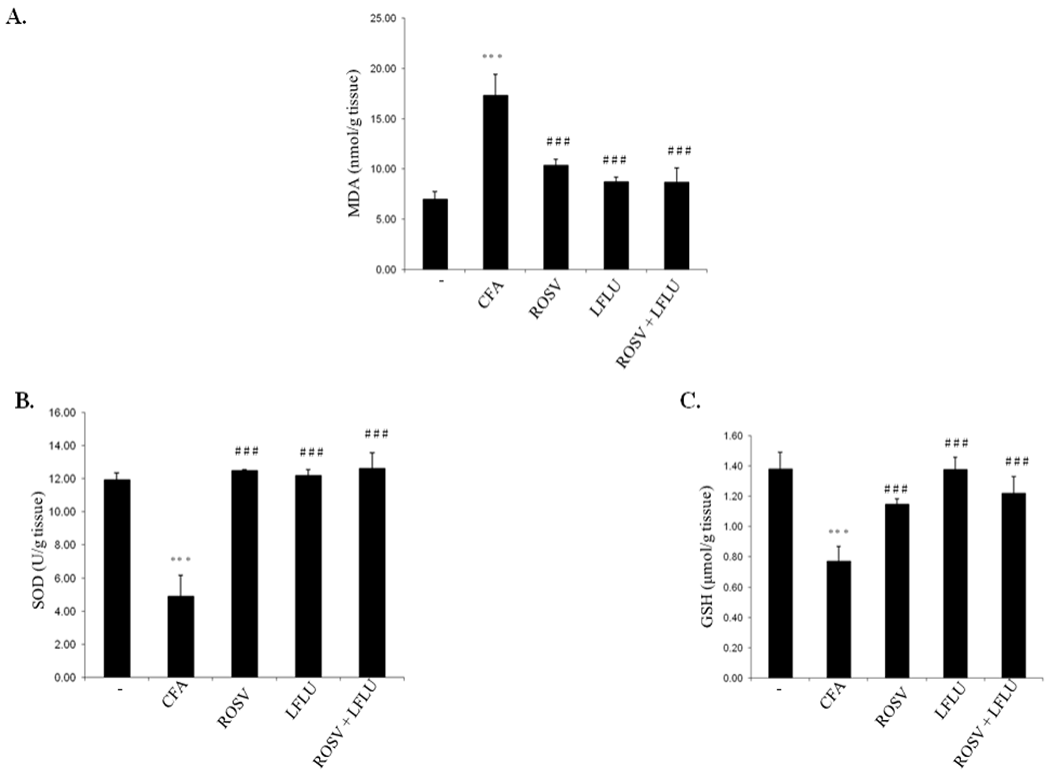
Effect of ROSV and/or LFLU on MDA and GSH content as well as SOD activity in experimental model of RA induced by CFA MDA (A) and SOD activity (B) as well as GSH content (C) in joint tissue of animals treated with with either vehicle (−) or CFA (0.4ml Complete Fruends Adjuvant S.C in right hind paw) or CFA+ROSV (10 mg/kg/day) or CFA+LFLU (10 mg/kg/day) or CFA+(ROSVand LFLU in half doses) were determined. Data represent means ± S.D. (n=8), *** p < 0.001 versus control, ### p < 0.001, versus CFA alone-treated animals

The by-product of lipid peroxidation MDA was highly increased in joint tissue of arthritic animals (Fig. 5A). On the other hand, administration of either ROSV or LFLU significantly reduced MDA in joint tissue of arthritic animals (Fig. 5A). Concomitant use of ROSV and LFLU in half doses significantly reduced the MDA level in joint tissue of arthritic rats (Fig. 5A).

### 3.6 ROSV and/or LFLU Increase GSH Content and SOD Activity in Joint Tissue of Arthritic Animals

As shown in Fig. 5B, SOD activity in arthritic animals was significantly reduced. However, administration of either ROSV or LFLU significantly increased SOD activity in arthritic rats. Concomitant use of ROSV and LFLU in half doses significantly increased SOD activity in arthritic animals. Also, GSH content in joint tissue of arthritic animals was highly reduced (Fig. 5C). On the other hand, treatment of arthritic rats with either ROSV or LFLU significantly increased the GSH content in joint tissue of arthritic animals (Fig. 5C). Concomitant use of ROSV and LFLU in half doses significantly increased GSH content in arthritic animals (Fig. 5C).

### 3.7 ROSV and/or LFLU Decrease Paw Size in Arthritic Animals

**Fig. 6.**
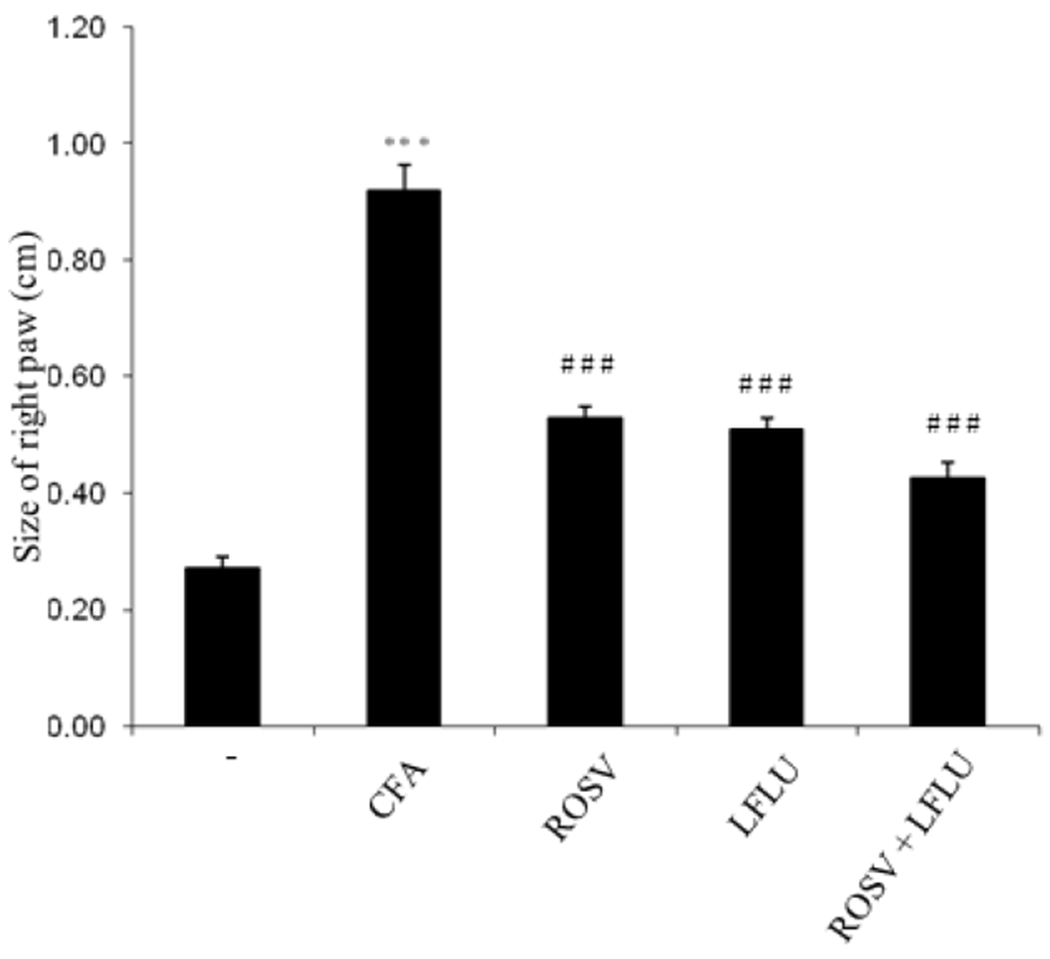
Effect of ROSV and/or LFLU on the size of right paw in experimental model of RA induced by CFA Size of paw from animals treated with with either vehicle (−) or CFA (0.4ml Complete Fruends Adjuvant S.C in right hind paw) or CFA+ROSV (10 mg/kg/day) or CFA+LFLU (10 mg/kg/day) or CFA+(ROSVand LFLU in half doses) was measured. Data represent means ± S.D. (n=8), *** p < 0.001 versus control, ### p < 0.001 versus CFA alone-treated animals.

Administration of CFA significantly induced paw in joint tissue as compared to control (Fig. 6). However, paw size was highly reduced in arthritic animals treated with either ROSV or LFLU (Fig. 6). Concomitant use of ROSV and LFLU in half doses significantly reduced paw size in arthritic animals (Fig. 6).

### 3.8 ROSV and/or LFLU Improved the Histopathological Alterations Induced by CFA in Joint Tissue

**Fig. 7.**
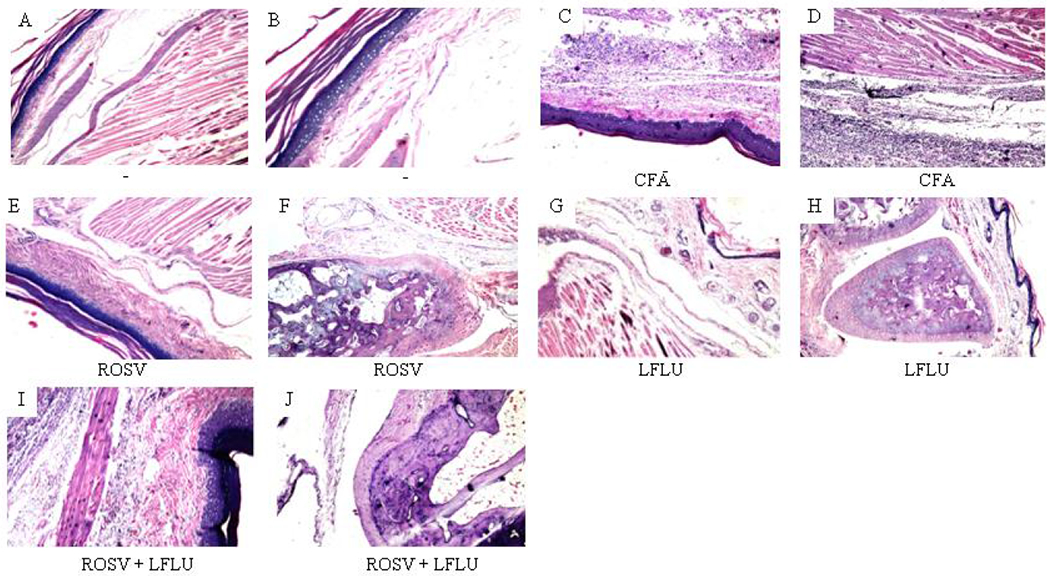
A. A section of the Paw of rat (control) showing normal histological feature of the skin (with epidermis and dermis) as well as the underlying subcutaneous tissue and musculature (H&E 16). B. A section of the Paw of rat (control) showing magnification of (A) to identify epidermis and dermis with subcutaneous tissue (H&E 40). C. A section of the Paw of arthritic rat showing epidermal acanthosis with massive infiltration of inflammatory cells in the dermis and underlying subcutaneous tissue (H&E 16). D. A section of the paw of arthritic rat showing inflammatory cells aggregation in subcutaneous tissue and extended to the muscular layer (H&E 16). E. A section of the paw of rat received ROSV showing normal histological structure of the skin layers, subcutaneous tissue and musculature (H&E 16). F. A section of the paw of rat received ROSV showing mild degeneration and mild atrophy in the articular cartilaginous surface (H&E 16). G. A section of the paw of rat received LFLU showing normal histological structure of the skin layers, subcutaneous tissue and musculature (H&E 16). H. A section of the paw of rat received LFLU showing normal histological feature of articular cartilaginous surface and synovial membrane (H&E 16). I. A section of the paw of rat received ROSV+ LFLU in half doses showing little hyperkeratosis and mild acanthosis in the epidermis with few infiltration of inflammatory cells in the deep dermis, subcutaneous tissue and musculature (H&E 16). J. A section of the paw of rat received ROSV+ LFLU in half doses showing little degeneration in the articular cartilaginous surface (H&E 16)

In contrast to control (Fig. 7A, 7B), treatment of rats with CFA significantly induced acanthosis in the epidermal layer associated with massive infiltration of inflammatory cells and aggregation in the subcutaneous tissue as well as the musculature (Fig. 7C and 7D). Interestingly, no histopathological alteration in the skin layers, subcutaneous tissue and musculature (Fig. 7E) was detected in arthritic rats treated with ROSV. Only mild degeneration was detected in the articular cartilaginous surface (Fig. 7F). Also, no histopathological alteration was detected in the skin layers, subcutaneous tissue, musculature (Fig. 7G), articular cartilaginous surface and synovial membrane (Fig. 7H) in arthritic animals treated with LFLU. Most importantly, only little hperkeratosis and mild acanthosis in the epidermis associated with few inflammatory cells infiltration in the deep dermis, subcutaneous tissue and musculature (Fig. 7I) as well as few degeneration in the cartilaginous articular surface (Fig. 7J) were detected in arthritic animals treated with both ROSV and LFLU in half doses.

## 4. DISCUSSION

Management of RA is a major health problem till now. The use of classical drugs in treatment of RA is limited due to their low safety. Therefore, it was interesting to find a new drug with high ability against inflammation and more safe. In the present study, CFA-induced rheumatoid arthritis model was used as it shares the human disease in various signs and symptoms [30]. In this study, induction of rheumatoid arthritis with CFA was found to be associated with a clear reduction in PPAR-α expression. Interestingly, treatment of rats with either ROSV or LFLU (standard DMARD) significantly induced PPAR-α expression in joint tissue of arthritic animals. Most importantly, concomitant administration of ROSV and LFLU in half doses significantly induced PPAR-α expression. This agree with previous study reported that ROSV has anti-inflammatory activity in PPARs-dependent manner [14]. Also, it has been shown that ROSV upregulates the expression of PPAR-α in vitro [31]. Interestingly, this increase in PPAR-α expression was associated with a significant reduction in NF-κB expression in arthritic animals treated with either ROSV or LFLU. These data are in line with previous study demonstrated that the antiinflammatory characters of ROSV may be attributed to its ability to inhibit NF-κB activity [4]. Most importantly, concomitant use of ROSV and LFLU in half doses significantly reduced NF-κB expression which plays an important role in the transcription of proinflammatory cytokines, MMPs, and iNOS [5]. Previously, it has been reported that the anti-inflammatory activity of statin may be attributed to its ability to inhibit iNOS expression [32,33]. In line with this study, iNOS expression was significantly reduced in arthritic animals treated with either ROSV or LFLU. Interestingly, concomitant use of ROSV and LFLU in half doses significantly inhibited iNOS expression in joint tissue of arthritic animals indicating that ROSV as well as LFLU has the ability to exert cells protection against CFA-induced nitrosative stress via inhibition of iNOS expression and subsequent reduction in NO level. Furthermore, the expression of MMP-9 and MMP-2 were highly induced in joint tissue of arthritic animals. In harmony with previous findings [34,35], treatment of arthritic rats with ROSV significantly reduced the expression of MMP-9 and MMP-2 in joint tissue. Also, LFLU administration significantly reduced the expression of MMP-9 and MMP-2 in arthritic animals. Importantly, administration of ROSV in combination with LFLU in half doses significantly reduced the expression of MMP-9 and MMP-2 in arthritic rats. Previously, it has been shown that TNF-α plays an important role in rheumatoid synovitis [36]. In the present findings, the serum level of TNF-α was highly increased in arthritic animals. Interestingly, TNF-α level was highly decreased in arthritic animals treated with either ROSV or LFLU. Similar findings were obtained in other studies [37,38]. Most importantly, concomitant use of ROSV and LFLU in half doses significantly decreased the TNF-α level. Also, IL-6 has been shown to display proinflammatory characters that are thought to be involved in the pathogenesis of RA [39]. The present work demonstrates that IL-6 level in serum of arthritic animals was highly increased. In agreement with other findings [35,40,41], administration of ROSV significantly decreased the serum IL-6 level in arthritic animals. The serum level of IL-6 was also decreased in arthritic rats treated with LFLU. Interestingly, concomitant use of ROSV and LFLU in half doses significantly decreased the serum IL-6 level in arthritic animals. The connection between oxidative stress and chronic inflammation is well known [42]. Oxidative stress is a condition in which the level of reactive oxygen species (ROS) increases overtime either by an increase in their production, decrease in the endogenous antioxidants and/or the combination of both [43]. Lipid peroxidation usually affects cell integrity [44]. The increase in the by-product of lipid peroxidation MDA in joint tissue of arthritic rats may reflect the oxidative stress. The present work shows that the by-product of lipid peroxidation MDA was highly increased in joint tissue of arthritic animals. Interestingly, administration of either ROSV or LFLU significantly reduced lipid peroxidation in joint tissue of arthritic animals. Most importantly, concomitant use of ROSV and LFLU in half doses significantly reduced lipid peroxidation in joint tissue of arthritic animals. These data are in line with previous finding demonstrated that ROSV has the ability to inhibit lipid peroxidation induced by piroxicam in gastric, liver, and kidney tissues [45]. Furthermore, SOD activity in arthritic animals was significantly reduced. In line with previous findings [46,47], administration of either ROSV or LFLU significantly increased SOD activity in arthritic group. Most interestingly, concomitant use of ROSV and LFLU in half doses significantly increased SOD activity in arthritic rats. Also, GSH content in joint tissue of arthritic animals was highly reduced. Interestingly, treatment of arthritic rats with either ROSV or LFLU significantly increased the GSH content in joint tissue of arthritic rats. These data are in agreement with previous findings [35,48]. Most importantly, concomitant use of ROSV and LFLU in half doses significantly increased GSH content in arthritic rats indicating that ROSV as well as LFLU has the ability to restore the antioxidant capacity in joint tissue of arthritic animals via enhancement of GSH content and SOD activity resulting in a great protection against oxidative stress induced by CFA. Furthermore, the present study shows that treatment of animals with CFA significantly induced paw in joint tissue compared with control. Importantly, paw size was significantly reduced in arthritic animals treated with either ROSV or LFLU. Most interestingly, concomitant use of ROSV and LFLU in half doses significantly reduced paw size in arthritic rats. Moreover, administration of either ROSV or LFLU as well as concomitant use of ROSV and LFLU in half doses significantly improved histopathological alteration in joint tissue of arthritic animals.

## 5. CONCLUSION

**Fig. 8.**
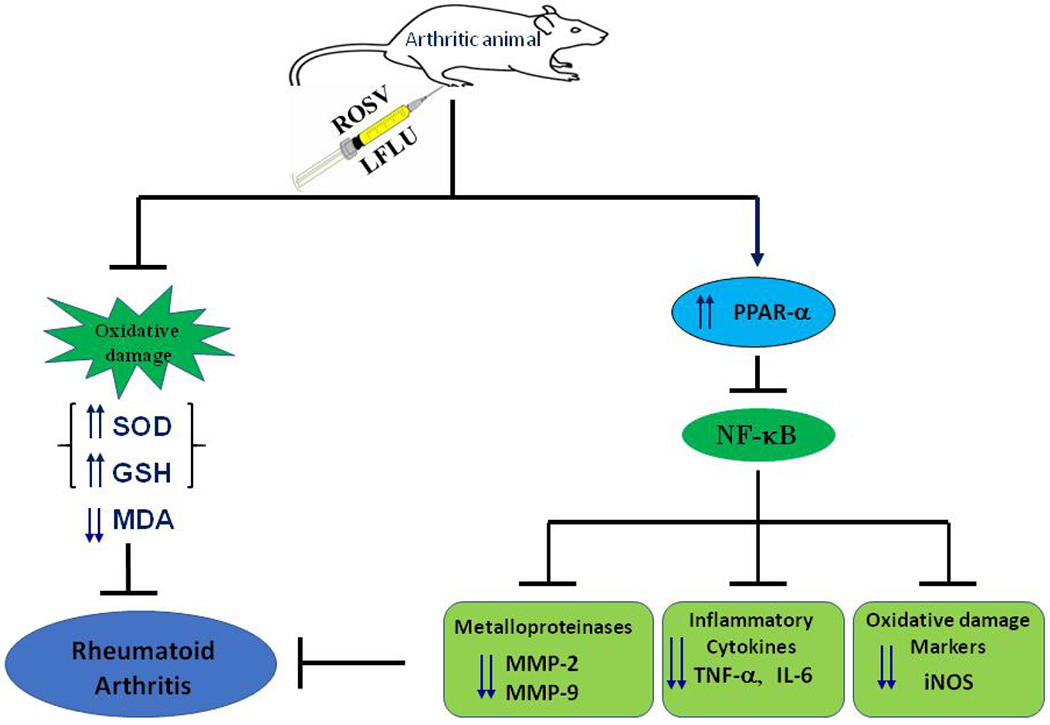
Schematic summary demonstrating the modulatory effect of either ROSV and/or LFLU on CFA-induced RA in experimental model

Our findings demonstrate that administration of either ROSV or LFLU or ROSV+LFLU (in half doses) inhibits RA in experimental model induced by CFA via induction of PPAR-α and subsequent inhibition of NF-κB resulting in a clear reduction in the inflammatory mediators (IL-6, TNF-α) and the matrix metalloproteinases (MMP-9, MMP-2) as well as iNOS expression (Fig. 8). Furthermore, oxidative stress was highly reduced in arthritic animals treated with either ROSV or LFLU or ROSV+LFLU (in half doses) as indicated by an increase in the endogenous antioxidants (SOD, GSH) and a clear reduction in the byproduct of lipid peroxidation MDA (Fig. 8). Taken together, this reduction in the inflammatory mediators and the balance between ROS production and the endogenous antioxidant defense system that was restored in joint tissues by either ROSV or LFLU or ROSV+LFLU (in half doses) are translated into a clear reduction in the size of right paw and improvement in the histopathological changes in arthritic animals. Finally, these data may support the concept of using the PPAR-α agonist ROSV as a valuable adjuvant in RA therapy. Furthermore, the use of both drugs (ROSV+LFLU) in half doses to manage RA may give similar effects that are usually obtained with the full doses and reduces the side effects that are usually caused by the full doses of these drugs. Further clinical studies are warranted to examine such an effect in human subjects.

## References

[1] Smolen JS, Aletaha D, Koeller M, Weisman MH, Emery P. New therapies for treatment of rheumatoid arthritis. Lancet. 2007;370:1861–1874. 17570481 10.1016/S0140-6736(07)60784-3

[2] Snaith ML. ABC of rheumatology: Gout, hyperuricaemia, and crystal arthritis. BMJ. 1995;310:521–524. 7888900 10.1136/bmj.310.6978.521 PMC2548885

[3] Guo Q, Wang Y, Xu D, Nossent J, Pavlos NJ, Xu J. Rheumatoid arthritis: Pathological mechanisms and modern pharmacologic therapies. Bone Res. 2018;6:15. 29736302 10.1038/s41413-018-0016-9 PMC5920070

[4] Okamoto H, Iwamoto T, Kotake S, Momohara S, Yamanaka H, Kamatani N. Inhibition of NF-kappaB signaling by fenofibrate, a peroxisome proliferator-activated receptor-alpha ligand, presents a therapeutic strategy for rheumatoid arthritis. Clin Exp Rheumatol. 2005;23:323–330. 15971419

[5] Tak PP, Firestein GS. NF-κB: A key role in inflammatory diseases. J Clin Invest. 2001;107:7–11. 11134171 10.1172/JCI11830 PMC198552

[6] Ricote M, Glass CK. PPARs and molecular mechanisms of transrepression. Biochim Biophys Acta. 2007;1771:926–935. 17433773 10.1016/j.bbalip.2007.02.013 PMC1986735

[7] Majdalawieh A, Ro HS. PPAR gamma1 and LXR alpha face a new regulator of macrophage cholesterol homeostasis and inflammatory responsiveness, AEBP1. Nucl recept signal. 2010;8:e004. 20419060 10.1621/nrs.08004 PMC2858268

[8] Chinetti G, Fruchart JC, Staels B. Peroxisome proliferator-activated receptors (PPARs): nuclear receptors at the crossroads between lipid metabolism and inflammation. Inflamm Res. 2000;49:497–505. 11089900 10.1007/s000110050622

[9] Kersten S, Desvergne B, Wahli W. Roles of PPARs in health and disease. Nature. 2000;405:421–424. 10839530 10.1038/35013000

[10] Delerive P, De Bosscher K, Besnard S, Vanden Berghe W, Peters JM, Gonzalez FJ Peroxisome proliferator-activated receptor alpha negatively regulates the vascular inflammatory gene response by negative cross-talk with transcription factors NF-kappaB and AP-1. J Biol Chem. 1999;274:32048–32054. 10542237 10.1074/jbc.274.45.32048

[11] Abeles AM, Pillinger MH. Statins as antiinflammatory and immunomodulatory agents: A future in rheumatologic therapy?. Arthritis Rheum. 2006;54:393–407. 16447216 10.1002/art.21521

[12] Shahin D, El Toraby DE, Abdel-Malek H, Boshra V, Elsamanoudy AZ, Shaheen D. Effect of peroxisome proliferator-activated receptor gamma agonist (pioglitazone) and methotrexate on disease activity in rheumatoid arthritis (experimental and clinical study). Clin Med Insights Arthritis Musculoskelet Disord. 2011;4:1–10. 21339857 10.4137/CMAMD.S5951 PMC3040074

[13] Bernatsky S, Hudson M, Suissa S. Anti-rheumatic drug use and risk of serious infections in rheumatoid arthritis. Rheumatology. 2007;46:1157–1160. 17478469 10.1093/rheumatology/kem076

[14] Kleemann R, Princen HMG, Emeis JJ, Jukema JW, Fontijn RD, Horrevoets AJG, Rosuvastatin reduces atherosclerosis development beyond and independent of its plasma cholesterol–lowering effect in APOE* 3-Leiden transgenic mice: evidence for antiinflammatory effects of rosuvastatin. Circulation. 2003; 108:1368–1374. 12939225 10.1161/01.CIR.0000086460.55494.AF

[15] Abdin AA, Abd El-Halim MS, Hedeya SE, El-Saadany AAE. Effect of atorvastatin with or without prednisolone on Freunds adjuvant induced-arthritis in rats. Eur J Pharmacol. 2012;676:34–40. 22197001 10.1016/j.ejphar.2011.11.052

[16] Funk JL, Chen J, Downey KJ, Clark RA. Bone protective effect of simvastatin in experimental arthritis. J Rheumatol. 2008;35:1083–1091. 18464303

[17] Manna SK, Aggarwal BB.Immunosuppressive leflunomide metabolite (A77 1726) blocks TNF-dependent nuclear factor-kappa B activation and gene expression. J Immunol. 1999;162:2095–2102. 9973483

[18] Li EK, Tam L, Tomlinson B. Leflunomide in the treatment of rheumatoid arthritis. Clin Ther. 2004;26:447–459. 15189743 10.1016/s0149-2918(04)90048-3

[19] Hamilton LC, Vojnovic I, Warner TD. A771726, the active metabolite of leflunomide, directly inhibits the activity of cyclo-oxygenase-2 in vitro and in vivo in a substrate-sensitive manner. Br J Pharmacol. 1999;127:1589–1596. 10455314 10.1038/sj.bjp.0702708 PMC1566153

[20] Snekhalatha U, Anburajan M, Venkatraman B, Menaka M. Evaluation of complete Freunds adjuvant-induced arthritis in a Wistar rat model. Z Rheumatol. 2013;72:375–382. 23208192 10.1007/s00393-012-1083-8

[21] Seif El-Din SH, El-Lakkany NM, El-Naggar AA, Hammam OA, Abd El-Latif HA, Ain-Shoka AA. Effects of rosuvastatin and/or β-carotene on non-alcoholic fatty liver in rats. Res Pharm Sci. 2015;10:275–287. 26600855 PMC4623617

[22] Breedveld FC, Dayer JM. Leflunomide: Mode of action in the treatment of rheumatoid arthritis. Ann Rheum Dis. 2000;59:841–849. 11053058 10.1136/ard.59.11.841 PMC1753034

[23] Zaitone SA, Abo-Gresha NM. Rosuvastatin promotes angiogenesis and reverses isoproterenol-induced acute myocardial infarction in rats: Role of iNOS and VEGF. Eur J Pharmacol. 2012;691:134–142. 22732653 10.1016/j.ejphar.2012.06.022

[24] Thoenes GH, Sitter T, Langer KH, Bartlett RR, Schleyerbach R. Leflunomide (HWA 486) inhibits experimental autoimmune tubulointerstitial nephritis in rats. Int J Immunopharmacol. 1989;11:921–929. 2613396 10.1016/0192-0561(89)90114-8

[25] Chong AS, Ma LL, Shen J, Blinder L, Yin DP, Williams JW. Modification of humoral responses by the combination of leflunomide and cyclosporine in Lewis rats transplanted with hamster hearts. Transplantation. 1997;64:1650–1657. 9422397 10.1097/00007890-199712270-00004

[26] Mounieb F, Ramadan L, Akool El-S, Balah A. Propolis alleviates concanavalin A-induced hepatitis by modulating cytokine secretion and inhibition of reactive oxygen species. Naunyn-Schmiedebergs Arch Pharmacol. 2017;390:1105–1115. 28761978 10.1007/s00210-017-1410-3

[27] Akool El-S. Molecular mechanisms of the protective role of wheat germ oil against cyclosporin A-induced hepatotoxicity in rats. Pharm Biol. 2015;53:1311–1317. 25858514 10.3109/13880209.2014.980584

[28] Hwang SJ, Kim JH, Shim JW, Kim DS, Jung HL, Park MS, Peroxisome proliferator-activated receptor-gamma expression in the lung tissue of obese rats. Yonsei Med J. 2011;52:495–501. 21488194 10.3349/ymj.2011.52.3.495 PMC3101042

[29] Gao Y, Yu W, Duan X, Ni L, Liu H, Zhao H, Wasp venom possesses potential therapeutic effect in experimental models of rheumatoid arthritis. Evid Based Complement Alternat Med. 2020;2020:6394625. 32328136 10.1155/2020/6394625 PMC7165351

[30] Suke SG, Negi H, Mediratta PK, Banerjee BD, Sharma KK. Anti-arthritic and anti-inflammatory activity of combined pioglitazone and prednisolone on adjuvant-induced arthritis. Eur J Pharmacol. 2013;718:57–62. 24075936 10.1016/j.ejphar.2013.09.019

[31] Sheng L, Ye P, Liu Y. Atorvastatin upregulates the expression of PPAR alpha/gamma and inhibits the hypertrophy of cardiac myocytes in vitro. Zhonghua xin xue guan bing za zhi. 2005;33:1080–1084. 16563274

[32] Tan W, Xue-bin C, Tian Z, Xiao-wu C, Pei-pei H, Zhi-bin C, Effects of simvastatin on the expression of inducible nitric oxide synthase and brain-derived neurotrophic factor in a lipopolysaccharide-induced rat model of Parkinson disease. Int J Neurosci. 2016;126:278–286. 26000813 10.3109/00207454.2015.1012758

[33] Ahmed YM, Messiha BAS, Abo-Saif AA. Protective effects of simvastatin and hesperidin against complete freunds adjuvant-induced rheumatoid arthritis in rats. Pharmacology. 2015;96:217–225. 26345515 10.1159/000439538

[34] Wang Y, Li H, Wang X, Yuan M, Li G. Combination of rosuvastatin and probucol inhibits MMP-9 expression via upregulation of miR-497 in cultured HUVECs and apoE knockout mice. J Thromb Thrombolysis. 2016;4:592–605. 10.1007/s11239-015-1291-6 26502925

[35] Guo H, Shi Y, Liu L, Sun A, Xu F, Chi J. Rosuvastatin inhibits MMP-2 expression and limits the progression of atherosclerosis in LDLR-deficient mice. Arch Med Res. 2009;40:345–351. 19766896 10.1016/j.arcmed.2009.07.006

[36] Choy EH, Panayi GS. Cytokine pathways and joint inflammation in rheumatoid arthritis. N Engl J Med. 2001;344:907–916. 11259725 10.1056/NEJM200103223441207

[37] Lee S, Lee Y, Kim J, An J, Kim K, Lee H, Atorvastatin and rosuvastatin improve physiological parameters and alleviate immune dysfunction in metabolic disorders. Biochem Biophys Res Commun. 2016;478:1242–1247. 27565724 10.1016/j.bbrc.2016.08.101

[38] Gómez-García A, Torres GM, Ortega-Pierres LE, Rodríguez-Ayala E, Alvarez-Aguilar C. Rosuvastatin and metformin decrease inflammation and oxidative stress in patients with hypertension and dyslipidemia. Rev Esp Cardiol. 2007;60:1242–1249. 18082089 10.1157/13113929

[39] Cicuttini FM, Byron KA, Maher D, Wootton AM, Muirden KD, Hamilton JA. Serum IL-4, IL-10 and IL-6 levels in inflammatory arthritis. Rheumatol Int. 1995;14:201–216. 7724996 10.1007/BF00262298

[40] Gualtero DF, Viafara-Garcia SM, Morantes SJ, Buitrago DM, Gonzalez OA, Lafaurie Gl. Rosuvastatin inhibits interleukin (IL)-8 and IL-6 production in human coronary artery endothelial cells stimulated with Aggregatibacter actinomycetemcomitans serotype b. J periodontal. 2017;88:225–235. 10.1902/jop.2016.160288 27739345

[41] Koufany M, Jouzeau J, Moulin D. Fenofibrate vs pioglitazone: Comparative study of the anti-arthritic potencies of PPAR-alpha and PPAR-gamma agonists in rat adjuvant-induced arthritis. Biomed Mater Eng. 2014;24:81–88. 24928921 10.3233/BME-140977

[42] Xie X, Li H, Wang Y, Wan Z, Luo S, Zhao Z, Therapeutic effects of gentiopicroside on adjuvant-induced arthritis by inhibiting inflammation and oxidative stress in rats. Int Immunopharmacol. 2019;76:105840. 31487614 10.1016/j.intimp.2019.105840

[43] Sá da Fonseca LJ, Nunes-Souza V, Goulart MOF, Rabelo LA. Oxidative stress in rheumatoid arthritis: what the future might hold regarding novel biomarkers and add-on therapies. Oxid Med Cell Longev. 2019;2019:7536805. 31934269 10.1155/2019/7536805 PMC6942903

[44] Dix TA, Aikens J. Mechanisms and biological relevance of lipid peroxidation initiation. Chem Res Toxicol. 1993;6:2–18. 8448344 10.1021/tx00031a001

[45] Abdeen A, Aboubakr M, Elgazzar D, Abdo M, Abdelkader A, Ibrahim S, Rosuvastatin attenuates piroxicam-mediated gastric ulceration and hepatorenal toxicity in rats. Biomed Pharmacother. 2019;110:895– 905. 30572194 10.1016/j.biopha.2018.11.004

[46] Yu S, Zhou X, Hou B, Tang B, Li J, Zhang B. Protective effect of rosuvastatin treatment by regulating oxidized low-density lipoprotein expression in a rat model of liver fibrosis. Biomed Rep. 2016;5:311–316. 27588174 10.3892/br.2016.722 PMC4998105

[47] Chen Z, Li S, Zhao W, Chen X, Wang X. Protective effect of co-administration of rosuvastatin and probucol on atherosclerosis in rats. Can J Physiol Pharmacol. 2014;92:797–803. 25203284 10.1139/cjpp-2014-0169

[48] Yu Y, Jin L, Zhuang Y, Hu Y, Cang J, Guo K. Cardioprotective effect of rosuvastatin against isoproterenol-induced myocardial infarction injury in rats. Int J Mol Med. 2018;41:3509–3516. 29568858 10.3892/ijmm.2018.3572

